# Influence of Flocculating Agents and Structural Vehicles on the Physical Stability and Rheological Behavior of Nitrofurantoin Suspension

**DOI:** 10.17795/jjnpp-12716

**Published:** 2014-05-03

**Authors:** Eskandar Moghimipour, Anayatollah Salimi, Saeed Rezaee, Maryam Balack, Somayeh Handali

**Affiliations:** 1 Cellular and Molecular Research Center, Ahvaz Jundishapur University of Medical Sciences, Ahvaz, IR Iran; 2 Nanotechnology Research Center, Ahvaz Jundishapur University of Medical Sciences, Ahvaz, IR Iran; 3 Department of Pharmaceutics, School of Pharmacy, Zanjan University of Medical Science, Zanjan, IR Iran; 4 Department of Pharmaceutics, Faculty of Pharmacy, Ahvaz Jundishapur University of Medical Sciences, Ahvaz, IR Iran

**Keywords:** Nitrofurantoin, Suspensions, Wetting Agents, Rheology

## Abstract

**Background:**

Nitrofurantoin is a nitrofuran antibiotic that has been used for treatment of urinary tract against positive and negative bacteria.

**Objectives:**

The aim of this study was to evaluate the effect of structural vehicles and flocculating agents on physical stability and rheological behavior of nitrofurantoin suspension.

**Materials and Methods:**

To formulate the suspensions, the effect of glycerin and polysorbate 80 as wetting agents was evaluated and their particle sizes were determined using the sieve method. Then to achieve controlled flocculation, sodium citrate and aluminum chloride were added. After choosing the suitable wetting and flocculating agents, structural vehicles such as sodium carboxyl methyl cellulose and Veegum were evaluated individually and in combination. In addition, the effect of sorbitol on density of continuous phase and some physical stability parameters such as sedimentation volume, degree of flocculation and ease of redispersion of the suspensions were evaluated. After incorporation of structural vehicles, the rheological properties of formulations were also determined to find their flow behavior.

**Results:**

According to the results, glycerin (0.2%) and sodium citrate (0.3%) had the best effect on the suspension stability as wetting and flocculating agents, respectively. Rheological properties of formulations showed pseudoplastic behavior with some degree of thixotropy.

**Conclusions:**

In conclusion, the suspension containing Veegum 1%, sodium carboxy methyl cellulose 1%, glycerine 0.2%, sodium citrate 0.3% and sorbitol 20 % was chosen as the most physically stable formulation.

## 1. Background

Nitrofurantoin is a synthetic nitrofuran that has been commonly used for treatment of urinary tract and gastrointestinal infections. Nitrofurantoin is commercially available as tablets or oral suspensions and parenteral liquid. The drug has numerous toxic effects such as pulmonary and hepatic toxicity. Nitrofurantoin is not able to penetrate body tissues well, so it should not be used for treatment of kidney or prostate infections (1-5). A suspension is a dispersed system in which the internal phase consists of solid particles and the external phase is a liquid vehicle. Suspensions are the best conventional dosage forms of drugs with slow dissolution rate and have patient compliance (6). A suspension should be uniform and any sedimentation which occurs during storage should be easily redispersed on agitation (7). Controlled flocculation and rheological modification of suspensions are important factors for their preparation. Flocculated suspensions are settled rapidly to form large loose and easily dispersable sediments (8). Rheological study of suspensions provides valuable information for efficient utilization, transport and handling of materials for industrial applications. The thixotropy and hysteresis loop are amongst the main rheological phenomena. Thixotropy is defined as isothermal comparatively slow recovery, on standing of a material, which has lost its consistency through shearing. The area between the ascending and descending curves, called the hysteresis loop, can give information about structure breakdown and rebuilding (9, 10).

## 2. Objectives

The aim of the present study was to evaluate the effect of structural vehicles and flocculating agents on physical stability and rheological behavior of nitrofurantoin suspension.

## 3. Materials and Methods

Glycerin, polysorbate 80, sorbitol, aluminum chloride and Veegum were purchased from Merck, Germany. Sodium citrate was obtained from Kia Behsam Chemie (K.B.C), IR Iran. Sodium carboxy methyl cellulose (NaCMC) and nitrofurantoin were purchased from NoviAnt, Sweden and Ubichem, England, respectively.

### 3.1. Preparation of Suspensions

Polysorbate 80 (0.2%) and glycerin (0.2%) were used as wetting agents. The concentration of nitrofurantoin was 0.5%. Their particle sizes were determined using sieves methods. Twenty-five milliliters of distilled water was added to the polysorbate and glycerin and this mixture was then gradually added to nitrofurantoin powder. The total volume of the suspension was 50 mL. Composition of different formulations of nitrofurantoin suspension including wetting agents and their particle sizes are shown in Table 1. The suspensions were stored at room temperature for the following evaluations.

**Table 1.** Composition of Nitrofurantoin Formulations Regarding the Amount of Wetting Agents And Their Particle Sizes

Formulation	Glycerin, %	Polysorbate 80, %	Particle Size, mesh
**F_1_**	0.2	-	-
**F_2_**	0.2	-	60
**F_3_**	0.2	-	100
**F_4_**	0.2	-	200
**F_5_**	0.2	-	230
**F_6_**	-	0.2	100
**F_7_**	-	0.2	200

To achieve controlled flocculation, different concentrations of sodium citrate (0.08, 0.1, 0.15, 0.2, 0.25, 0.3, 0.5, 0.9, 1.2 and 1.6%) and aluminum chloride (0.05, 0.08 and 0.1%) were added to the suspensions. For decreasing of sedimentation volume of the suspension, sorbitol with concentrations of 15% and 20% was used. By comparison of their sedimentation volume and improvement of their appearance, suitable wetting and flocculating agents were chosen and then different structural vehicles were added. The effect of Veegum (0.5, 1, 1.5 and 2%), NaCMC (0.25, 0.5 and 1 %.), and their combination as structural vehicles was evaluated.

### 3.2. Physical Stability

#### 3.2.1. Sedimentation Volume

After preparation, the suspensions were kept in a graduated cylinder and their sedimentation volume was measured daily. The heights of sediments were measured when there was no change in three consecutive readings. Their sedimentation volumes were calculated using the following formula; F = V_u_/V_o_,

where F is sedimentation volume, and V_u_ and V_o_ are volume of sediment and volume of suspension, respectively.

#### 3.2.2. The Degree of Flocculation

Unflocculated 0.5% nitrofurantoin suspension was prepared in the absence of wetting and flocculating agents and stored at room temperature. When the sedimentation volume remained unchanged, the degree of flocculation was calculated using the following formula; β = F/F_∞_,

where β is the degree of flocculation, and F and F_∞_ are sedimentation volume of flocculated and deflocculated suspensions, respectively.

#### 3.2.3. The Ease of Redispersion

The suspension samples were placed in glass cylinders and rotated periodically around 360 degrees until thorough dispersion was achieved. The number of rotations needed for complete redispersion was recorded. Redispersion was evaluated by visual observation (N).

#### 3.2.4. Rheological Assessment

The rheological behavior of nitrofurantoin suspensions in different formulations (Table 2) was determined using a Brookfield viscometer (model LVDL-I + digital, USA with No. 3 spindle). The viscosity of samples was determined at 0.5, 1, 2, 2.5, 4, 5, 10, 20, 50 and 100 rpm after a one-minute rotation at room temperature. The results were plotted as rheograms and their rheological behavior was determined by fitting on the corresponding Newtonian and non-Newtonian equations. Also, the presence and extent of thixotropy were calculated by calculating the area between ascending and descending curves using the trapezoidal rule.

**Table 2.** Composition of Nitrofurantoin Suspensions Prepared for Rheological Assessment

	Veegum, %	NaCMC, %	Sorbitol, %	Sodium Citrate, %	Glycerin, %
**F_8_** ** (control) ** ^**a**^	-	-	-	-	-
**F_9_**	1	1	15	0.3	0.2
**F_10_**	1	1	20	0.3	0.2
**F_11_**	0.5	1	20	0.3	0.2
**F_12_**	1.5	1	20	0.3	0.2

^a^ Water as vehicle and no excipient was added.

### 3.3. Statical Methods

Kruskal-Wallis test followed by Conover-Inman test for multiple-comparison were performed to compare sedimentation volumes, the degrees of flocculation and the ease of redispersion. For all analyses, statistical significance was assessed at a level of 0.05 using analysis. The data are shown as the median and range of at least three measurements.

## 4. Results

Supernatant of suspensions containing glycerin had relatively clear appearance in comparison with suspensions containing polysorbate 80. Except formulations F_4_ and F_5_, the sediments in suspension containing glycerin were dispersed easily in comparison with suspensions containing polysorbate 80. Particles size is an important factor that affects the solubility and stability of suspensions. High sedimentation volume was observed in formulations F_4_ and F_5_ that had used sieving size of 200 and 230 meshes, respectively and showed no significant difference with other formulations of suspension containing glycerin (P > 0.05). Regarding their ease of redispersion due to formation of cake, formulations F_4_ and F_5_ were significantly different from other formulations containing glycerin (P < 0.05). This phenomenon may be occurring due to increase of contact surface and accumulation of particles that results from particles size decrease. Different formulations of suspensions containing polysorbate 80 showed no significant change in their ease of redispersion and sedimentation volume (P > 0.05). According to median values of F, N and b for nitrofurantoin suspension in different formulations of glycerin and polysorbate 80 (Table 3), glycerin was selected as the most suitable wetting agent. Supernatant of suspensions containing different concentrations of sodium citrate were completely clear, while they were turbid for suspensions containing aluminum chloride. Flocculation in suspensions improved with increasing concentration of sodium citrate. The presence of 0.3% sodium citrate caused the highest sedimentation volume, although by adding the concentration of sodium citrate, the sedimentation volume decreased. Deflocculation may occur in the suspension due to change in zeta potential by adding a concentration higher than 0.3%. Aluminum chloride as a flocculating agent did not show desirable properties, because in comparison with sodium citrate, the sedimentation speed was decreased and their supernatant was unclear. Therefore, according to median values of F, N and b (Table 4), the formulation containing sodium citrate 0.3% as a flocculating agent was significantly better than the other formulations (P < 0.05). Also the results indicated that nitrofurantoin might have a positive charge, because by using aluminum chloride, which is a cationic compound, flocculation did not occur. However, sodium citrate as an anionic electrolyte may cause flocculation.

**Table 3.** The Median (Range) of F and N for Nitrofurantoin Suspension in Different Formulations Containing Glycerin and Polysorbate 80 ^a^

Formulation	Median	
	F	N
**F_1_**	0.08 ± 0.015	5 ± 2
**F_2_**	0.12 ± 0.03	3 ± 3
**F_3_**	0.18 ± 0.02	4 ± 4
**F_4_**	0.2 ± 0.02	40 ± 2
**F_5_**	0.25 ± 0.02	60 ± 10
**F_6_**	0.02 ± 0.005	40 ± 0
**F_7_**	0.07 ± 0.05	40 ± 0

^a^ Data are presented in Mean ± Range.

**Table 4.** The Median (Range) of F, N and b for Nitrofurantoin Suspension in Formulations Containing Sodium Citrate and Aluminum Chloride as Flocculating Agents ^a^

Median	Formulation		
	F	N	β
**Sodium citrate 0.08%**	0.05 ± 0.01	4 ± 2	1 ± 0.2
**Sodium citrate 0.1%**	0.07 ± 0.015	4 ± 1	1.4 ± 0.3
**Sodium citrate 0.15%**	0.08 ± 0.05	3 ± 1	1.6 ± 0.1
**Sodium citrate 0.2%**	0.095 ± 0.015	2 ± 1	1.9 ± 0.3
**Sodium citrate 0.25%**	0.11 ± 0.02	2 ± 1	2.2 ± 0.4
**Sodium citrate 0.3%**	0.25 ± 0.08	1 ± 1	5 ± 1.6
**Sodium citrate 0.5%**	0.08 ± 0.02	3 ± 2	1.6 ± 0.3
**Sodium citrate 0.9%**	0.07 ± 0.04	4 ± 1	1.4 ± 0.9
**Sodium citrate 1.2%**	0.067 ± 0.05	4 ± 3	1.4 ± 0.9
**Sodium citrate 1.6%**	0.09 ± 0.02	4 ± 1	1.8 ± 0.4
**Aluminum chloride 0.05%**	0.05 ± 0.01	5 ± 2	1 ± 0.2
**Aluminum chloride 0.08%**	0.06 ± 0.01	4 ± 3	1.1 ± 0.1
**Aluminum chloride 0.1%**	0.08 ± 0.01	5 ± 2	1.6 ± 0.2

^a^ Data are presented in Mean ± Range.

Median values of F, N and b for nitrofurantoin suspension in different structural vehicles are shown in Table 5. The suspension containing Veegam 1.5% had the highest sedimentation volume and degree of flocculation, although its ease of redispersion was low. Also, the supernatant of the suspension was clear and it was easily dispersed. The suspension containing NaCMC showed a significant differece in its sedimentation volume, degree of flocculation and ease of redispersion compared with suspensions containing Veegam (P < 0.05), and the supernatant was unclear and slowly redispersed. According to the results, for combination of Veegam and NaCMC, the prosperity of suspension was better. Suspensions containing Veegam 1%, NaCMC 0.5% and Veegam 1%, NaCMC 1% showed no significant difference in their sedimentation volume and ease of redispersion, but showed a signification difference in their degree of flocculation. Therefore, the suspension containing Veegam 1% and NaCMC 0.5% was selected as the best formulation.

**Table 5.** Median (Range) of F, N and b for Nitrofurantoin Suspension in Different Structural Vehicles a

Formulation	Median		
	F	N	β
**Veegam 0.5%**	0.32 ± 0.04	2 ± 2	6.40 ± 0.80
**Veegam 1%**	0.49 ± 0.04	3 ± 1	9.80 ± 0.80
**Veegam 1.5%**	0.66 ± 0.03	2 ± 1	13.20 ± 0.60
**Veegam 2%**	0.42 ± 0.04	3 ± 1	8.40 ± 0.80
**NaCMC 0.25%**	0.24 ± 0.04	3 ± 1	4.80 ± 1.20
**Veegam 0.5%, NaCMC 0.5%**	0.90 ± 0.06	2 ± 2	18 ± 0.8
**Veegam 1%, NaCMC 0.5%**	0.97 ± 0.02	3 ± 1	19.40 ± 0.44
**Veegam 1.5% , NaCMC 0.5%**	0.96 ± 0.40	3 ± 2	19.20 ± 0.40
**Veegam 2%, NaCMC 0.5%**	0.96 ± 0.01	4 ± 1	19.10 ± 0.20
**Veegam 1%, NaCMC 1%**	0.95 ± 0.01	2 ± 1	19.00 ± 0.23

^a^ Data are presented in Mean ± Range.

Median values of F, N and b for nitrofurantoin suspension containing the combination of different structural vehicles and flocculating agents are shown in Table 6. According to the results, the suspension containing Veegam 1%, NaCMC 0.5% and sodium citrate 0.3% had the highest sedimentation volume and degree of flocculation, while its ease of redispersion was less and showed a significant difference in its properties in comparison with the other formulations (P < 0.05). Combination of Veegam and NaCMC improved physical stability and appearance of the samples containing the combination.

**Table 6.** Median (Range) of F, N and b for Nitrofurantoin Suspension of Different Structural Vehicles and Flocculating Agents ^a^

Formulation	Median		
	F	N	β
**Veegam 0.5%, sodium citrate 0.3%**	0.49 ± 0.02	2 ± 1	9.8 ± 0.4
**Veegam 1%, sodium citrate 0.3%**	0.96 ± 0.04	2 ± 1	19.20 ± 0.8
**Veegam 1.5%, sodium citrate 0.3%**	0.66 ± 0.02	1 ± 1	13.20 ± 0.4
**Veegam 2%, sodium citrate 0.3%**	0.46 ± 0.02	2 ± 1	9.20 ± 0.4
**NaCMC 0.25%, sodium citrate 0.3%**	0.36 ± 0.06	2 ± 1	7.2 ± 1.20
**Veegam 0.5%, NaCMC 0.5%, sodium citrate 0.3%**	0.97 ± 0.03	2 ± 1	19.40 ± 0.60
**Veegam 1%, NaCMC 0.5%, sodium citrate 0.3%**	0.99 ± 0.01	1 ± 1	19.80 ± 0.20
**Veegam 1.5%, NaCMC 0.5%, sodium citrate 0.3%**	0.97 ± 0.02	2 ± 1	19.40 ± 0.30
**Veegam 2%, NaCMC 0.5%, sodium citrate 0.3%**	0.97 ± 0.02	2 ± 1	19.40 ± 0.28
**Veegam 1%, NaCMC 1%, sodium citrate 0.3%**	0.99 ± 0	1 ± 1	19.76 ± 0.10
**NaCMC 0.5%, sodium citrate 0.3%**	0.70 ± 0.06	2 ± 1	14 ± 0.80

^a^ Data are presented in Mean ± Range.

**Figure 1.**
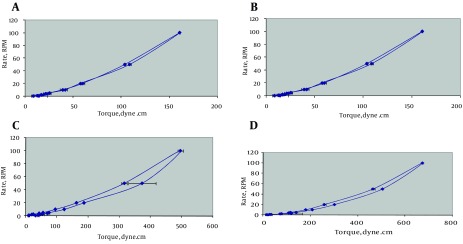
Rheograms and Thixotropy of Nitrofurantoin Suspensions in a Vehicle Containing A) F9; B) F10; C) F11; and D) F12 are showing their rheological behaviors and thixotropies.

For reducing the sedimentation volume of the suspension, sorbitol with concentrations of 15% and 20% was used. Results showed that sorbitol (20%) had the best effect on reducing sedimentation volume. Results of rheologic study showed that all of the formulations had pseudoplastic behavior with some degree of thixotropy (Figure 1). The values of N as an indicator for the type of flow for different formulations are presented in Table 7. An important parameter for predicting flow behavior of liquid dispersion is the area of hysteresis loop between the ascending and descending curves of the rheogram, which are shown in Table 7. Evaluation of hysteresis area revealed that all of the formulations except formulation F_10_ and F_13_ had positive thixotropy behavior. It is generally accepted that the greater the hysteresis area, the stronger the thixotropic property and a good suspension should have high thixotropy and pseudoplastic behavior (11).

**Table 7.** Amount of N for Evaluation of Rheological Behavior and Median (Range) of Hysteresis Loop of Different Formulations ^a^

Formulation	N	Hysteresis Loop
**F_8_**	1.979	165.47 ± 198.05
**F_9_**	1.426	-1481.52 ± 107.98
**F_10_**	1.684	2413.19 ± 234.61
**F_11_**	2.023	-3152.16 ± 134.68
**F_12_**	3.747	2807.22 ± 500.5

^a^ Data are presented in Mean ± Range.

Regarding the results of sedimentation volume and the ease of redispersion, suspension containing Veegam 1% and NaCMC 0.5% was chosen as the best formulation. However, the results of the rheological study showed that these formulations (F_9_ and F_11_) had antithixotropy behavior. Thus, formulation F_10_ was selected as the best formulation. It is suggested that low concentration of NaCMC may affect the rheological behavior of suspensions and the highest improving effect may be achievied by using higher concentrations of NaCMC.

## 5. Discussion

Rheological properties of suspensions depend on their particle size, shape and surface modification (12). Viriyaroj et al. during 2009 reported that carboxymethylcellulose, methylcellulose and xanthan gum used as suspending agents caused desirable rheological behavior. Xanthan gum exhibits plastic or pseudoplastic flow and this behavior was possibly a result of shearing action on the long chain molecule of xanthan gum (13). Gallardo et al. in 2006 investigated rheological properties of ethylcellulose latex. The results showed that viscoelastic behavior was affected by temperature. High temperature also caused a change in particle shape (14). Moreria et al. in 2010 investigated influence of oleic acid on the rheology and *in vitro* release of lumiracoxib from poloxamer gels. The results of the rheological study showed a pseudoplastic behavior, and an increase of poloxamer from 20% to 30% w/w and oleic acid increased the viscosity of the gel (15).Khunawattanakul et al. in 2008 investigated the rheology, flocculate size and zeta potential of the chitosan, a positively charged polymer, and magnesium aluminum silicate, the negatively charged clay dispersions.The results showed that the electrostatic interaction between chitosan and magnesium aluminum silicate caused a change in flow behavior and flocculation of the composite dispersions, depending on the high and low molecular weights of chitosan. Increasing of hysteresis loop of the composite dispersion was found when magnesium aluminum silicate was added, which increased their thixotropic properties. Heat treatment also caused a decrease in viscosity and hysteresis loop of high molecular weights of chitosan-magnesium aluminum silicate dispersion. The decrease in viscosity of the composite dispersion might have been caused by a reduction of intra-molecular hydrogen bonding of chitosan and a decrease in hydrogen-bonded hydration of chitosan, while heating did not affect the hydration of magnesium aluminum silicate, because magnesium aluminum silicate dispersions were prepared and hydrated using hot water in the preparation process (16). Li et al. in 2005 reported that hydroxyethyl cellulose in combination with bromododecane increased viscosity and caused thermal stability (17).

The combination of glycerin 0.2% and sodium citrate 0.3% showed the best wetting and flocculating properties. Rheological studies showed pseudoplastic behavior. In conclusion, in different formulations of nitrofurantoin suspension regarding their F, N and β, the suspension containing Veegum 1%, sodium carboxy methyl cellulose 1%, glycerin 0.2%, sorbitol 20 % and sodium citrate 0.3% was the most physically stable formulation and showed the most proper rheological behavior.
